# Administration of parenteral nutrition during therapeutic hypothermia: a population level observational study using routinely collected data held in the National Neonatal Research Database

**DOI:** 10.1136/archdischild-2020-321299

**Published:** 2021-05-05

**Authors:** Chris Gale, Dusha Jeyakumaran, Nicholas Longford, Cheryl Battersby, Shalini Ojha, Kayleigh Oughham, Jon Dorling

**Affiliations:** 1 Neonatal Medicine, School of Public Health, Imperial College London, London, UK; 2 School of Medicine, University of Nottingham, Derby, UK; 3 Neonatal Unit, University Hospitals of Derby and Burton NHS Foundation Trust, Derby, UK; 4 Neonatal Data Analysis Unit, Imperial College London Faculty of Medicine, London, UK; 5 Division of Neonatal—Perinatal Medicine, Dalhousie University—Faculty of Medicine, Halifax, Nova Scotia, Canada

**Keywords:** epidemiology, neonatology, statistics

## Abstract

**Background:**

Parenteral nutrition is commonly administered during therapeutic hypothermia. Randomised trials in critically ill children indicate that parenteral nutrition may be harmful.

**Objective:**

To examine the association between parenteral nutrition during therapeutic hypothermia and clinically important outcomes.

**Design:**

Retrospective, population-based cohort study using the National Neonatal Research Database; propensity scores were used to create matched groups for comparison.

**Setting:**

National Health Service neonatal units in England, Scotland and Wales.

**Participants:**

6030 term and near-term babies, born 1/1/2010 and 31/12/2017, who received therapeutic hypothermia; 2480 babies in the matched analysis.

**Exposure:**

We compared babies that received any parenteral nutrition during therapeutic hypothermia with babies that did not.

**Main outcome measures:**

Primary outcome: blood culture confirmed late-onset infection; secondary outcomes: treatment for late onset infection, necrotising enterocolitis, survival, length of stay, measures of breast feeding, hypoglycaemia, central line days, time to full enteral feeds, discharge weight.

**Results:**

1475/6030 babies (25%) received parenteral nutrition. In comparative matched analyses, the rate of culture positive late onset infection was higher in babies that received parenteral nutrition (0.3% vs 0.9%; difference 0.6; 95% CI 0.1, 1.2; p=0.03), but treatment for presumed infection was not (difference 0.8%, 95% CI −2.1 to 3.6, p=0.61). Survival was higher in babies that received parenteral nutrition (93.1% vs 90.0%; rate difference 3.1, 95% CI 1.5, 4.7; p<0.001).

**Conclusions:**

Receipt of parenteral nutrition during therapeutic hypothermia is associated with higher late-onset infection but lower mortality. This finding may be explained by residual confounding. Research should address the risks and benefits of parenteral nutrition in this population.

**What is already known on this topic?**

- There is little evidence to inform nutritional practice during and after therapeutic hypothermia for babies with hypoxic ischaemic encephalopathy.
- Parenteral nutrition is commonly administered to term and near-term babies during therapeutic hypothermia.
- A randomised trial in paediatric critical care found delayed provision of parenteral nutrition beyond 7 days superior to early parenteral nutrition in term babies.

**What this study adds?**

- Approximately one in four babies in the UK receive parenteral nutrition during therapeutic hypothermia and this proportion is increasing.
- Parenteral nutrition is associated with more late onset blood stream infection and also with higher survival.
- Randomised trials evaluating neurodevelopment and validated measures of infection are needed to determine the risks and benefits of parenteral nutrition in babies receiving therapeutic hypothermia.

## Background

Optimal nutrition for term and near-term babies receiving therapeutic hypothermia is uncertain. Although administration of parenteral nutrition to this population is not recommended by UK national guidance relating to therapeutic hypothermia^1^ or parenteral nutrition,^2^ national survey data suggest that this practice is common.^3^

Potential benefits of parenteral nutrition, rather than intravenous dextrose and electrolytes, include improved brain growth and repair with theoretical neurodevelopmental benefit. However, putative advantages must be balanced against accumulating evidence of harm, such as increased incidence of infection, from early parenteral nutrition from randomised trials of critically ill children.^4^

We aimed to identify an optimal approach to parenteral nutrition for infants receiving therapeutic hypothermia. Key outcomes such as blood-stream infection are rare in this population, consequently a randomised controlled would need to be very large. We therefore undertook an observational study using routinely recorded clinical data and applying propensity score matching to form groups for comparison with near-identical distributions of background variables.

Our primary aim was to assess the association between parenteral nutrition administered during therapeutic hypothermia and the incidence of late-onset blood stream infection; predefined secondary outcomes were also examined.

## Methods

We undertook a retrospective cohort study using routinely recorded clinical data held in the National Neonatal Research Database (NNRD). The study was prospectively registered (ISRCTN47404296; NCT03278847) and the protocol published.^5^ We applied propensity score methodology to form matched subgroups of infants with similar background characteristics that either received parenteral nutrition during therapeutic hypothermia or did not. We compared the rates of late-onset infection and other outcomes between these two matched groups.

The data source was the NNRD,^6^ which holds data from infants admitted to National Health Service (NHS) neonatal units in England, Scotland and Wales (approximately 90 000 infants annually). In the UK, therapeutic hypothermia is not provided outside of NHS neonatal units. Data are extracted from neonatal electronic health records completed by health professionals during routine clinical care. A defined data extract, the Neonatal Dataset,^7^ of approximately 450 data items, is transmitted quarterly, cleaned, and duplicates and queries about discrepant and implausible data are fed back to health professionals and rectified. Completeness and accuracy of NNRD data have been validated.^6^ A patient-level dataset was extracted from the NNRD for the purposes of this analysis. Data linkage was not performed.

The a priori sample size was 1500 pairs of babies, estimated to detect (two-sided significance 5%, power 90%) a difference of 2% in late-onset infection assuming a 3% rate of infection in the parenteral nutrition group.

We extracted data from infants born 1/1/2008 to 31/12/2017 and admitted to neonatal units contributing to the NNRD in England, Scotland and Wales. All NHS neonatal units have contributed data to the NNRD since 2012 in England and Wales, and since 2015 in Scotland. Data were extracted for the duration of an infant’s neonatal stay.

Infants were included if they had a recorded gestational age of $\geq 36^{+0}$ weeks^+days^ at birth and were recorded as having received therapeutic hypothermia for 3 days or died during this 3-day period after receiving therapeutic hypothermia. Babies who had missing data for receipt of therapeutic hypothermia on the second day of hypothermia but who were recorded as receiving therapeutic hypothermia on both the preceding and following day and who did not die during cooling, had therapeutic hypothermia data for the second day imputed. No other data imputation was performed. Receipt of parenteral nutrition was defined as receiving parenteral nutrition of any type, by any route, for at least one day during therapeutic hypothermia. Codes used to define analysis groups are available in online supplemental table 1.

10.1136/archdischild-2020-321299.supp1 Supplementary data

The primary outcome was late-onset blood stream infection using the National Neonatal Audit Programme case definition.^8^ Secondary outcomes included suspected infection (five consecutive days of antibiotic treatment starting after day 3), severe necrotising enterocolitis (NEC) using the UK Neonatal Collaborative NEC study^9^ definition, pragmatically defined NEC (a recorded diagnosis of NEC and five or more consecutive days of antibiotics while nil by mouth), survival to discharge, length of neonatal unit stay, hypoglycaemia (diagnosed during neonatal unit stay), breast feeding at discharge, onset of breast feeding, day of first maternal milk, central line days, duration of parenteral nutrition, and weight for age SD score (SDS) at neonatal discharge. Details are given in online supplemental table 3.

To address potential confounders (eg, infants with multisystem disease may be more likely to not receive parenteral nutrition and to have poorer outcomes), we used propensity matching to form two subgroups of infants with similar background characteristics, including clinical condition and treatment when therapeutic hypothermia was started. The variables in the propensity model included: infant sex; maternal factors (age, ethnicity, deprivation decile); pregnancy factors (smoking status, multiplicity, duration of rupture of membranes, fever, suspected chorioamnionitis, hypothyroidism, diabetes, parity); infant factors (mode of delivery, gestational age, birth weight SDS, 1 min and 5 min Apgar score, chest compressions during resuscitation, emergency resuscitation drugs, intubated at resuscitation, umbilical cord gas values, time to first spontaneous breath); condition on admission prior to therapeutic hypothermia (blood pressure, glucose, heart rate, oxygen saturation, temperature); culture positive early-onset infection; treatment on day one (inotropes, mechanical ventilation, inhaled nitric oxide) and organisational factors (postnatal transfer within 24 hours, neonatal network of birth); details are given in online supplemental table 2. Neonatal services in the UK are organised in managed neonatal networks that comprise a small number of tertiary units in each network and a larger number of non-tertiary units that generally transfer infants to tertiary centres for ongoing therapeutic hypothermia.

Analyses applied the potential outcomes framework and propensity score methodology.^10^ We performed 1:1 matching of babies that received and did not receive parenteral nutrition during therapeutic hypothermia. For each infant, the propensity of the exposure (parenteral nutrition or no parenteral nutrition) was estimated by logistic regression that included all background variables as covariates, and a selection of their interactions. Matched pairs were formed within these groups. Pairs were first matched using birth year (four 2-year bands) and arterial umbilical cord pH (three bands: >7.0, 6.9–7.0, <6.9), 12 groups in total. Matched pairs were then formed within propensity score deciles defined separately for each background group; details are given in online supplemental material.

Outcomes in the resulting two matched subgroups were then compared and relative risks of outcomes derived. The SE of the estimate of the treatment effect was obtained by combining the within and between-replication SEs.^11^ All p values are two-sided. Analyses were performed using SAS V.9.4 (SAS Institute, Cary, North Carolina, USA) and R.^12^ To prevent potential identification of individuals where low numbers of events occurred, low counts are presented as <5.

Pre-planned sensitivity analyses:

1. Restricted to babies born 2012–2017 in England and Wales to determine whether less complete data prior to 2012 introduces bias.
2. Restricted to infants where all parenteral nutrition data were actively recorded, excluding infants missing nutrition data during the first 4 days.

A third, posthoc, sensitivity analysis was undertaken with the agreement of the Study Steering Committee to examine the impact of enteral nutritional practice on day one: enteral feeding on the first day of life was added as a background variable to the propensity model.

Prior to comparative analyses, it became clear that the proportion of missing data was high in 2008 and 2009; analysis was therefore restricted to babies born between 2010 to 2017.

A multiprofessional investigator group which included a parent of a baby that received therapeutic hypothermia and a parent representative designed and oversaw the study. Study outcomes reflected those prioritised as important by parents, patients and professionals.^13^ The study was overseen by an independent Study Steering Committee who approved all deviations from the original protocol.

## Results

Between 1/1/2010 and 31/12/2017, 703 907 babies were admitted to NHS neonatal units in England, Scotland or Wales; 6030 were ≥36 weeks gestational age and treated with therapeutic hypothermia for 3 days or died during treatment. Of these, 1475 babies (24.5%) received parenteral nutrition during therapeutic hypothermia, and this proportion increased slightly over time (linear slope=0.007, p=0.003).

Thirty babies (30/6030, 0.5%) had a pure growth of a recognised pathogen in a blood culture after day 3; 1559 babies (25.5%) had late onset infection when defined as five consecutive days of antibiotics. Survival to discharge was high (90.3%, 5444 babies) and median length of neonatal stay was 11 days (IQR 8–16). Almost all babies (5640, 93.6%) had a central line and the median duration was 5 days (IQR 3–6); 1208 babies (20.0%) had hypoglycaemia recorded at any point during their neonatal stay.

**Figure 1:**
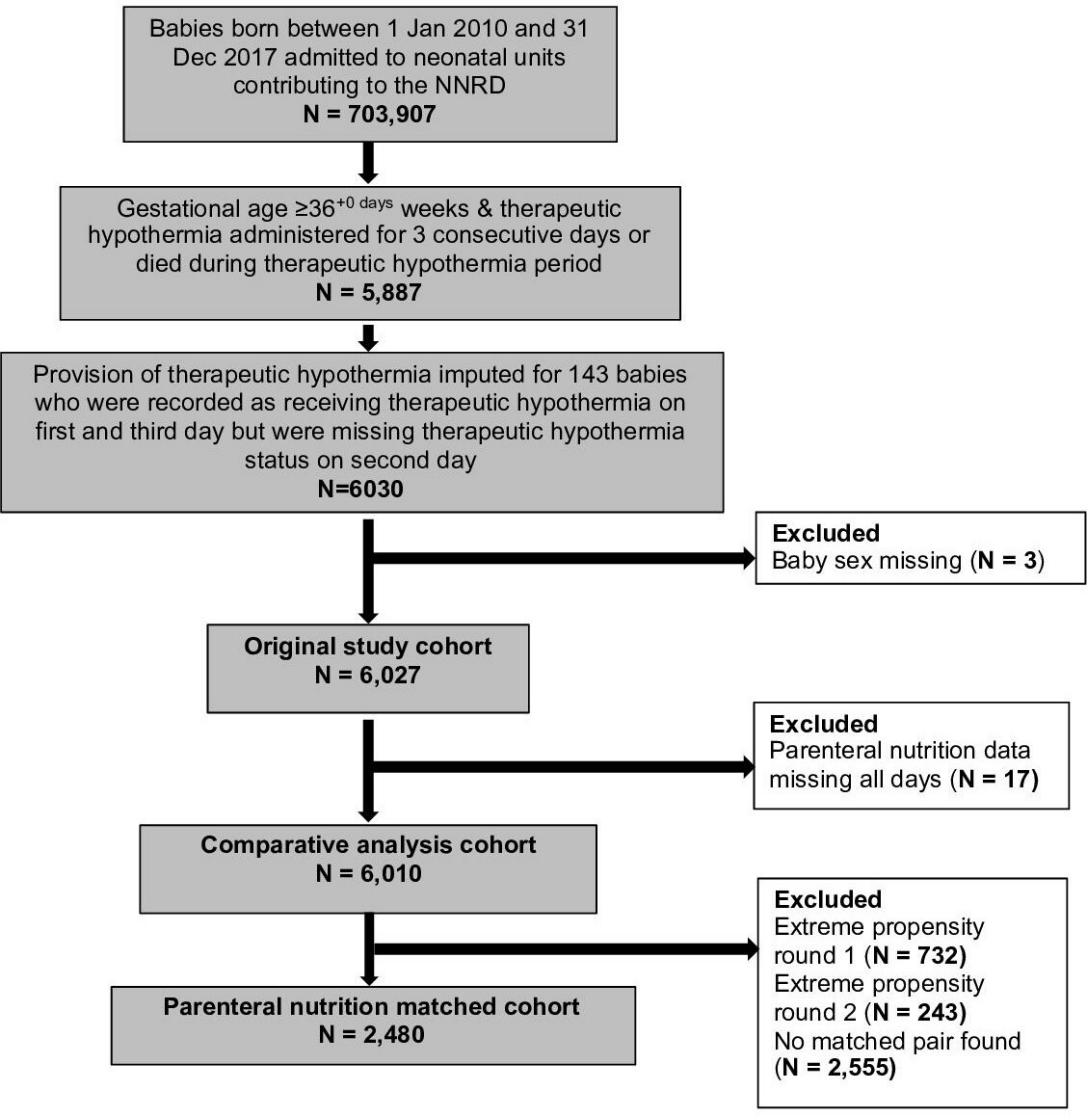
Participant flow through the study for the primary analysis. N, number; NNRD, National Neonatal Research Database.

Propensity matching was used to form two subgroups of 1240 infants with similar background characteristics for comparative analyses (figure 1). Good balance was achieved for all recorded background variables (table 1). In matched analyses, the incidence of blood culture confirmed that late onset infection was very low in both groups but higher in babies that received parenteral nutrition compared with babies that did not (11 (0.9%) vs <5 (0.3%); rate difference 0.6% (0.1 to 1.2), p=0.03). Pragmatically defined late onset infection was similar in babies who did (323, 26.1%) and did not receive parenteral nutrition (313, 25.3%) (rate difference 0.8% (−2.1 to 3.6), p=0.61) (table 2).

**Table 1:** Background variables by group: unmatched and matched cohorts

Variable	Unmatched cohort		Matched cohort	
	No parenteral nutrition	Parenteral nutrition	No parenteral nutrition	Parenteral nutrition
N	4535	1475	1240	1240
Male				
N (%)	2507 (55.3)	810 (54.9)	652 (52.6)	664 (53.5)
Gestational age at birth (weeks)				
Mean (SD)	39.4 (1.6)	39.4 (1.6)	39.4 (1.6)	39.4 (1.6)
Birth weight (g)				
Mean (SD)	3385 (621)	3321 (631)	3330 (609)	3328 (628)
Caesarean delivery				
N (%)	2066 (47.7)	667 (47.1)	545 (45.9)	549 (46.1)
Maternal age				
Median (IQR)	30 (26–35)	31 (26–34)	30 (26–34)	31 (26–34)
Maternal suspected chorioamnionitis				
N (%)	479 (12.8)	175 (14.5)	147 (14.5)	150 (14.6)
Smoking in pregnancy				
Yes N (%)	520 (13.3)	211 (16.9)	175 (16.6)	176 (16.8)
Missing N (%)	620 (15.8)	227 (18.2)	187 (17.8)	189 (18.0)
Ethnicity (maternal)				
White, %	65.7	61.4	80.8	79.9
Asian and Mixed, %	11.2	6.8	7.9	7.5
Black and Mixed, %	7.5	4.3	4.3	5.0
Other and missing, %	15.5	27.5	6.9	7.6
Maternal diabetes				
N (%)	191 (4.2)	65 (4.4)	49 (4.0)	53 (4.3)
Maternal deprivation score				
In deciles 1 or 2, %	27.9	22.3	23.9	21.7
Primiparous				
N (%)	2425 (53.5)	778 (52.7)	669 (54.0)	671 (54.1)
Umbilical cord arterial pH				
>7.0, N (%)	1439 (44.4)	451 (44.0)	396 (45.3)	396 (45.3)
6.9–7.0, N (%)	756 (23.3)	248 (24.2)	198 (22.7)	198 (22.7)
<6.9, N (%)	1049 (32.3)	326 (31.8)	280 (32.0)	280 (32.0)
Missing N (%)	1291 (28.5)	450 (30.5)	366 (29.5)	366 (29.5)
Apgar 5 min				
0–1 (%)	730 (16.1)	218 (14.8)	188 (15.1)	182 (14.7)
2–4 (%)	1704 (37.6)	563 (38.2)	450 (36.2)	470 (37.9)
5–7 (%)	1372 (30.3)	433 (29.4)	389 (31.3)	362 (29.2)
8–10 (%)	374 (8.2)	130 (8.8)	107 (8.6)	116 (9.4)
Missing (%)	355 (7.8)	131 (8.9)	107 (8.6)	109 (8.8)
Received chest compressions at resuscitation				
N (%)	1705 (37.6)	523 (35.5)	431 (34.8)	427 (34.4)
Received resuscitation drugs				
N (%)	719 (15.9)	206 (14.0)	176 (14.2)	172 (13.9)
Intubated at resuscitation				
N (%)	2925 (64.5)	935 (63.4)	784 (63.2)	783 (63.1)
Mechanical ventilation on day of admission				
N (%)	3508 (80.2)	1122 (79.5)	951 (79.6)	956 (80.2)
Treatment with inotropes on day of admission				
N (%)	1126 (26.0)	335 (23.9)	295 (25.0)	287 (24.2)

N, number.

**Table 2:** Outcomes by feeding group: unmatched and matched cohorts

Variable	Unmatched cohort		Matched cohort	
	No parenteral nutrition	Parenteral nutrition	No parenteral nutrition	Parenteral nutrition
N	3975	1872	1240	1240
Blood culture positive late onset infection				
N (%)	16 (0.4)	14 (0.9)	<5 (<0.5)	11 (0.9)
Late onset infection (pragmatic definition)				
N (%)	1175 (25.9)	383 (26.0)	313 (25.3)	323 (26.1)
Severe NEC (confirmed at surgery, postmortem or recorded on death certificate)				
N	6 (0.1)	<5 (<0.5)	7 (0.6)	<5 (<0.5)
NEC (pragmatic definition)				
N (%)	52 (1.1)	16 (1.1)	17 (1.4)	13 (1.1)
Survival at discharge				
N (%)	4056 (89.5)	1374 (93.2)	1116 (90.0)	1154 (93.1)
Hypoglycaemia				
N (%)	946 (20.9)	258 (17.5)	235 (18.9)	212 (17.1)
Onset of breast feeding (days)				
Median (IQR)	7 (6–9)	7 (6–9)	7 (6–9)	7 (6–9)
Breast feeding at discharge				
N (%)	2110 (46.5)	670 (45.4)	582 (47.0)	575 (46.4)
Time to first mother’s milk (days)				
Median (IQR)	5 (4–6)	5 (3–5)	5 (4–6)	5 (3–5)
Days with a central venous line in situ				
Median (IQR)	5 (3, 6)	5 (4, 7)	5 (3, 6)	5 (4, 7)
Weight Z-score at discharge				
Median (IQR)	−0.6 (−1.4 to 0.2)	−0.7 (−1.4 to 0.1)	−0.7 (−1.4 to 0.1)	−0.6 (−1.4 to 0.2)
Length of stay (days)				
Median (IQR)	11 (8–17)	10 (7–13)	11 (8–16)	11 (8–16)

N, number; NEC, necrotising enterocolitis.

Survival to discharge was higher in babies that received parenteral nutrition (1154, 93.1%) compared with those that did not (1116, 90.0%) (rate difference 3.1% (1.5 to 4.7), p<0.001). The incidence of pragmatically defined necrotising enterocolitis was low and similar in babies that received parenteral nutrition (13 cases, 1.1%) and those that did not (17 cases, 1.4%) (rate difference (95% CI) −0.3% (−1.0 to 0.4), p=0.39). The duration in time that a baby had a central line in situ was higher in babies that received parenteral nutrition (6.0 days) versus those that did not (5.1) (difference 0.9 days (0.5 to 1.2), p<0.001) (table 3). Placement of a central venous line was common in both groups (92.3% of babies that did not receive parenteral nutrition and 97.9 of those that did). Measures of breast feeding, incidence of recorded hypoglycaemia, weight for gestation SDS at neonatal unit discharge and length of neonatal unit stay were all similar between babies that received parenteral nutrition and those that did not (tables 2 and 3). These findings were robust to sensitivity analyses (online supplemental table 4).

**Table 3:** Analysis of outcome variables for babies received vs did not receive parenteral nutrition

Variable	Intervention		Difference (95% CI)	OR estimate (95% CI)	P value
	No parenteral nutrition (95% CI)	Parenteral nutrition (95% CI)			
N	1240	1240			
Blood culture positive late onset infection, %	0.3 (0.1 to 0.5)	0.9 (0.4 to 1.4)	0.6 (0.1 to 1.2)	3.04 (0.95 to 9.76)	0.03*
Late onset infection (pragmatic definition), %	25.3 (23.6 to 27.1)	26.1 (23.8 to 28.3)	0.8 (−2.1 to 3.6)	1.04 (0.87 to 1.25)	0.61
NEC (pragmatic definition), %	1.4 (0.9 to 1.9)	1.1 (0.6 to 1.6)	−0.3 (−1.0 to 0.4)	0.77 (0.38 to 1.58)	0.39
Hypoglycaemia, %	19.0 (17.5 to 20.6)	17.0 (15.1 to 18.9)	−2.1 (−4.5 to 0.4)	0.87 (0.71 to 1.07)	0.10
Survival at discharge, %	90.0 (88.8 to 91.2)	93.1 (91.8 to 94.4)	3.1 (1.5 to 4.7)	1.50 (1.17 to 2.01)	<0.001
Breast feeding at discharge, %	47.0 (45.1 to 48.9)	46.4 (43.9 to 48.9)	−0.6 (−3.8 to 2.6)	0.98 (0.83 to 1.14)	0.71
Length of stay, days	14.1 (13.6 to 14.7)	15.0 (14.1 to 15.8)	0.8 (−0.2 to 1.8)		0.12
First suckling at breast, days	8.4 (8.0 to 8.7)	8.6 (8.0 to 9.1)	0.2 (−0.5 to 0.8)	–	0.56
First maternal milk, days	4.9 (4.8 to 4.9)	4.6 (4.5 to 4.8)	−0.2 (−0.4 to 0.1)	–	0.01
Duration of CV line, days	5.1 (5.0 5.3)	6.0 (5.7 to 6.3)	0.9 (0.5 to 1.2)	–	<0.001
Weight Z-score	−0.66 (−0.71 to 0.61)	−0.65 (−0.71 to –0.58)	0.02 (−0.07 to 0.10)	–	0.68

Results averaged over the 25 replications of the matching procedure.

CV, central venous; N, number; NEC, necrotising enterocolitis; NNAP, National Neonatal Audit Programme.

## Discussion

One in four babies that receive therapeutic hypothermia in the UK receive concurrent parenteral nutrition. After extensive matching across a comprehensive number of background variables, we find that culture confirmed blood stream infection and survival were both higher in babies that received parenteral nutrition. Other short-term morbidities and measures of breast feeding were not materially different between babies that did and did not receive parenteral nutrition. The propensity score methodology used in this study can only address imbalances in observed confounders, so residual confounding by indication cannot be excluded. Optimal parenteral nutrition for babies receiving therapeutic hypothermia is not known and randomised evaluations comparing early versus delayed parenteral nutrition, able to assess short-term and long-term outcomes, are required.

Despite widespread use, there are few data describing parenteral nutrition in babies during therapeutic hypothermia. Case studies report electrolyte disturbances in such infants receiving parenteral nutrition^14 15^ but we know of no published comparative data in this population. Randomised trials have compared early and delayed parenteral nutrition in critically ill adults^16^ and children,^4^ demonstrating beneficial outcomes with delayed commencement of parenteral nutrition. The latter, paediatric intensive care based, PEPaNIC trial reports data from 209 babies randomised to early (<24 hours) or late (>7 days) commencement of parenteral nutrition in a preplanned secondary analysis^17^: neonates with surgical or cardiac conditions that did not receive therapeutic hypothermia. This subgroup analysis found higher rates of infection in babies recruited below 1 week of age randomised to early parenteral nutrition, consistent with our results. However, and in contrast to our data, the PEPaNIC trial found no difference in mortality between early and delayed parenteral nutrition groups, although the sample size was low for such outcomes. The PEPaNIC trial also found a higher incidence of hypoglycaemia in neonates randomised to receive later parenteral nutrition which was not replicated in our study; this may be explained by diagnoses in the NNRD being linked with an ‘episode’ of care on a neonatal unit and not attributed to a specific day. Consequently, the temporal relationship between hypoglycaemia and parenteral nutrition cannot be directly examined. Evidence to support benefits of parenteral nutrition in this population is also limited. A putative benefit is improved brain growth, repair and consequent neurodevelopment; while this has not been directly evaluated, supplemental nutrition following brain injury shows promise.^18^ Considering the limited available neonatal data, the risks and benefits of parenteral nutrition in babies undergoing therapeutic hypothermia are clinically potentially important but highly uncertain.

This study was not a randomised evaluation of parenteral nutrition; however, we applied multiple approaches to limit bias: using multiple background data items, we formed matched groups for comparison. We followed a detailed, preregistered protocol that specified exposures, background factors and outcomes and the data items used to define them as well as the matching process.^5^ To examine whether data completeness influenced results, we undertook sensitivity analyses which led to similar findings to the main analysis. In addition, the use of routinely recorded data captured during clinical care reduced the risk of ascertainment bias, as data collection occurred well in advance of study conception. Use of such data facilitated inclusion of a large number of infants—the study sample was several times larger than all previous randomised trials of therapeutic hypothermia combined.

In this non-randomised study, we used propensity scores to form matched groups for analysis; the main limitation of this approach is that it can account only for measured confounders. Although a wide range of background and day-one data items were used to undertake matching and acceptable balance was obtained, we cannot exclude residual confounding or confounding from differences in unmeasured factors. This is likely to overestimate the benefit of parenteral nutrition: the ‘sickness’ of a baby may be more evident to clinicians than can be discerned from data held in the NNRD, and sicker babies may be both less likely to be commenced on parenteral nutrition and to survive. As with any study that uses routinely recorded data, completeness and accuracy are variable; this is particularly relevant to culture positive blood stream infection which has been found to be under-reported previously.^8^ However, data that form the NNRD are primarily entered as part of a baby’s clinical care, are used for purposes including national audit, funding and staffing and have been validated against independent clinical trial data.^6^ Results should be interpreted cautiously in light of these limitations.

These data and other published evidence suggest that parenteral nutrition may be associated with both harms and benefits in term and near-term babies who receive therapeutic hypothermia. Randomised controlled trials are required to elucidate whether the increasing use of parenteral nutrition in this group is beneficial. The low incidence of culture positive infection and low mortality confirm that a trial adequately powered to address such outcomes is unlikely to be feasible. Therefore, a future trial should focus on other validated measures of late-onset infection and neurodevelopmental outcomes with higher background rates that would make a randomised trial powered to detect a meaningful difference in such outcomes practicable.

## Conclusion

The use of parenteral nutrition is relatively common in babies who are undergoing therapeutic hypothermia and appears to be increasing. Optimal parenteral nutritional support for babies receiving therapeutic hypothermia could not be established in this large observational study.

## Data Availability

Data may be obtained from a third party through the National Neonatal Research Database with relevant approvals; more information is available here: www.imperial.ac.uk/neonatal-data-analysis-unit/neonatal-data/utilising-the-nnrd/.
